# Comparing Angular and Curved Shapes in Terms of Implicit Associations and Approach/Avoidance Responses

**DOI:** 10.1371/journal.pone.0140043

**Published:** 2015-10-13

**Authors:** Letizia Palumbo, Nicole Ruta, Marco Bertamini

**Affiliations:** 1 Department of Psychology, Liverpool Hope University, Liverpool, United Kingdom; 2 Department of Psychological Sciences, University of Liverpool, Liverpool, United Kingdom; 3 School of Art and Design, Cardiff Metropolitan University, Cardiff, United Kingdom; University of Manchester, UNITED KINGDOM

## Abstract

Most people prefer smoothly curved shapes over more angular shapes. We investigated the origin of this effect using abstract shapes and implicit measures of semantic association and preference. In Experiment 1 we used a multidimensional Implicit Association Test (IAT) to verify the strength of the association of curved and angular polygons with danger (safe vs. danger words), valence (positive vs. negative words) and gender (female vs. male names). Results showed that curved polygons were associated with safe and positive concepts and with female names, whereas angular polygons were associated with danger and negative concepts and with male names. Experiment 2 used a different implicit measure, which avoided any need to categorise the stimuli. Using a revised version of the Stimulus Response Compatibility (SRC) task we tested with a stick figure (i.e., the manikin) approach and avoidance reactions to curved and angular polygons. We found that RTs for approaching vs. avoiding angular polygons did not differ, even in the condition where the angles were more pronounced. By contrast participants were faster and more accurate when moving the manikin towards curved shapes. Experiment 2 suggests that preference for curvature cannot derive entirely from an association of angles with threat. We conclude that smoothly curved contours make these abstract shapes more pleasant. Further studies are needed to clarify the nature of such a preference.

## Introduction

The curve line has been described as the expression of grace and beauty [1]. Possibly this is the reason why it is extensively used to represent objects in many art contexts from visual art, products design and architecture. For example, [2] showed that car design has moved towards more rounded shapes across the last decades. This goes hand in hand with recent empirical research in aesthetics showing that observers prefer shapes with a curved contour line as compared to an angular contour line [3, 4, 5, 6]. This preference occurred with familiar or meaningful stimuli as well as with abstract geometrical shapes. Interestingly, preference for curvature over angularity is cross-cultural, it is not only specific to humans [7] and it is present at early stages of the development [8]. Despite the fact that the phenomenon is very powerful, the nature of the liking response for curved shapes is still under examination. Importantly preference for curved stimuli has been mostly examined through explicit evaluations.

In the current study we employed two different implicit measures to investigate the affective dimension underlying preference for abstract shapes. To this aim we designed irregular polygons with different contours (angular vs. curved), but matched in all other aspects. These stimuli were novel and abstract, avoiding possible interaction effect with valence, familiarity or semantic meaning that might mask the effect of the curvature [9].

### What makes curvature to be preferred?

There are different propositions for why observers tend to like curved shapes. On one hand, there are studies suggesting that observers prefer curved shapes over angular ones because the spiky transitions in an angular contour conveys a sense of threat. Therefore, the liking response for curvature could be a by-product of the dislike for angularity. The threat hypothesis for angular stimuli is supported by a bilateral activation of the amygdala, which is typically involved in the processing of threat [10, 11, 12]. Bar and coll. reported this activation with pairs of photographs depicting everyday objects or meaningless novel patterns [10]. The items in each pair differed exclusively in terms of contour (curved or angular). Larson and coll. found similar results with downward-pointing V shapes [12].

On the other hand there is evidence for a direct preference of smooth curves. [13] reported an fMRI study on the impact of curvature on aesthetic judgments in architecture. The authors used coloured pictures of internal spaces with curvilinear or rectilinear appearance. Participants gave two-forced choices responses: “beautiful” or “not beautiful” for the aesthetic dimension and “enter” or “exit” for the approach avoidance dimension. The curvilinear spaces were evaluated as more beautiful than the rectilinear ones. Moreover, the curvilinear spaces activated the anterior cingulate cortex, which responds to emotional aspects of stimuli and reward. Surprisingly, curvature did not affect the approach-avoidance decision.

Another evidence in favour of the liking for curvature can be found in [4, 5]. The authors compared patterns of coloured lines with angles to parabolic curves and straight lines (without angles or curves). The rationale of the study was that if preference for curvature is a by-product of the dislike for angularity, as angles might signal a threat, then also straight lines should be preferred, as they do not contain a threatening element. Conversely, if curvature is pleasant in itself it should be the most preferred pattern and should be liked more than both angular and straight lines. The latter prediction was confirmed suggesting that smooth curvature is more appealing than angular or straight lines.

### Explicit vs. implicit measures to study preference for curvature

Preference for curved stimuli so far has been investigated by asking participants to make explicit evaluations, either as a yes/no decision or using rating scales. Therefore, although exposure in some studies was short (for example 85ms) [3], an explicit evaluation is likely to include high-level cognitive processes and conscious decision-making.

In the current study we employed two different implicit measures to examine the relation between preference for curvature and affective processes. Specifically, we examined implicit associations between shapes (curved or angular) and other dimensions (as for example Safety: “safe” or “dangerous” and Valence: “positive” or “negative”) with the Implicit Association Test (IAT) [14]. In the past it has been shown that curved and angular shapes can be implicitly associated with sounds like ‘maluma’ and ‘takete’ respectively as well as with pleasant and unpleasant concepts [15, 16, 17]. In the same fashion we reasoned that Femininity is another logical category to examine, as it might be associated with curvature. Therefore, we included Gender as a third dimension. This would document the complex set of associations between categories.

Next, with the Stimulus Response Compatibility (SRC) task revised [18] using a stick figure (i.e., the manikin) we tested whether these implicit associations would determine congruent approach/avoidance reactions to smoothly curved and to angular shapes.

Overall these two experiments would (1) inform about the impact of semantic/affective processes in the liking formation for abstract shapes, (2) clarify to what extent the threat hypothesis related to angles is a plausible explanation for the preference of curved elements, (3) reveal whether different measures of implicit affective responses provide similar results and if such results match those with explicit evaluations.

### The current study: the use of the IAT and the SRC task revised to examine preference for curvature

In Experiment 1 we used the IAT, a tool that probes associations between stimulus pairs by asking participants to classify all stimuli. The IAT has been subjected to extensive methodological scrutiny [19] and recently it has been used in empirical aesthetics as an indirect measure of preference [20, 21, 22, 23, 24]. In order to describe the IAT procedure here we refer to the best-known IAT experiment [14]. Participants used two buttons to classify four stimulus categories. On some trials they saw pictures of either flowers or insects, and had to press one button for flower and the other button for insect. On interleaved trials, they saw either positive words (e.g., “love”) or negative words (e.g., “hate”). They had to press one button for positive, and the other for negative. In *congruent blocks*, the same button was used for “flower” or “love”, and the other was used for “insect” or “hate”. In *incongruent blocks*, the response mapping was reversed (so one button was used for “flower” and “hate”, the other was used for “insect” and “love”). Typically the task is more difficult (longer RTs and more errors) in the *incongruent* trials because people tend to associate flowers with positive objects and insects with negative objects and not the reverse. The difference on the RTs between congruent and incongruent blocks reflects an implicit association between the stimulus pairs. Following the same procedure in Experiment 1 we examined the strength of implicit associations of curved and angular polygons with other concepts (i.e. words associated with “safe” vs. “danger”). We expected curved polygons to be associated with “safe” words and angular polygons to be associated with “danger” words. This would account for the effect of implicit semantic associations on the liking evaluation.

In Experiment 2 we employed a different implicit measure that would be more informative about which one of the two associations is more likely to determine the preference for curvature. The SRC task-R (revised), as we call it here, tested whether the association of curved polygons with positive concepts and the association of angular polygons with negative concepts would determine a congruent approach/avoidance reaction. The original version of this task, called SRC task [18], consists of pressing one key as soon as possible to move a figure towards a stimulus and another to move it away.

In another version, called Manikin task, the authors used three key presses to give the impression to the observer that the manikin was walking [25]. Despite this difference in both these versions the responses of ‘approach’ and ‘avoidance’ are defined in terms of reaction time needed to decide and react (RTs on first key press). In our previous studies we adopted a new approach [4, 5], which combines some methodological aspects of the original task [18] and of its manipulation [25]. More specifically, first participants needed to press a button in order to see the manikin on the screen. In this way the affective mapping effects obtained were dependent on the activation of abstract, symbolic representations of the self and the objects [25]. Participants were instructed to move the manikin following the original procedure of the SRC task [18], but we did adopt the three-key press response as in the Manikin task [25]. We made this choice because although the manikin is just a stick figure it was designed in a way that while participants were pressing the same key three times they got the impression that he was walking, thus adding some ecological validity to the task. In terms of data analysis in the current SCR task-R we took into account two measures. The first one is the RT on the first key press (RT1), which reflects the time elapsed between the appearance of the stimulus and the first key press. The second is the RT on the third key press (RT3) given by the time elapsed between the appearance of the stimulus and the third key press (when the manikin stopped closed or away from the shape). As such, RT3 expresses the combination of simple RT and the time required to completing the ballistic movement of response execution [26, 27]. These two measures are informative in respect to (1) how fast the participants decided the direction of the movement (response selection: approach or avoidance) and (2) how fast they kept the manikin going in that direction. The first application of our SCR task-R, where only RT3 was analysed, showed a stronger tendency to approach curved polygons than to avoid angular polygons [4, 5]. However, the shapes employed did not vary for the amount of angularity and roundness. In the current work we applied the same task as in [4, 5] but we added polygons with more pronounced vertices to strictly examine whether an avoidance reaction would occur when shapes assume a spikier appearance. In conclusion, Experiment 2 will reveal whether implicit responses of approach/avoidance to contour match explicit evaluations [13].

## Experiment 1: Implicit associations of shapes with concepts

Implicit associations of curvature with other semantic dimensions can mediate preference evaluations. Therefore in Experiment 1 we wanted to test the existence of these implicit associations. We employed a “multidimensional IAT” [21] with 3 dimensions: IAT 1 for the “Danger” dimension (safe vs. dangerous words), IAT 2 for the “Valence” dimension (positive vs. negative words) and IAT 3 for the “Gender” dimension (female vs. male names). We added “Gender” as the third dimension because curved shapes, due to their roundness, might resemble the female body. Therefore female physical attributes might be evaluated more positively than male attributes because they contain more rounded elements. Because a person’s name conveys the information about the gender of the person we assumed that a female person’s name would be automatically associated with a female body, and that a male person’s name would be automatically associated with a male body. We predicted that curved shapes would be associated with words expressing safety, with positive valence and with female names. In contrast angular shapes would be associated with words expressing danger, with negative valence and with male names.

### Method

#### Participants

Twenty-four participants took part in the experiment (age range: 19–46, average age 21.5 years, 15 females). All participants had normal or corrected to normal vision. They provided a written consent for taking part and received course credits. The experiment was approved by the Ethics Committee of the University of Liverpool and was conducted in accordance with the Declaration of Helsinki (2008).

#### Stimuli and apparatus

**Fig 1:**
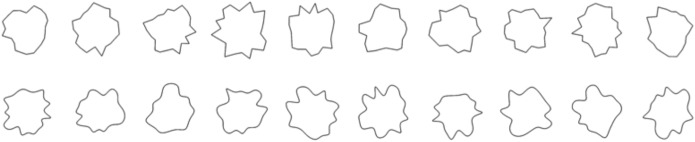
Stimuli in Experiment 1. The first row shows the ten polygons used for the category angular, and the bottom row shows the ten smoothed stimuli used for the category curved.

Stimuli consisted of irregular abstract shapes with a black contour line. Stimuli and experiment were created using Python and Psychopy [28]. The stimuli were generated starting from polygons that were based on sampling 22 vertices along the circumference of a circle. A full explanation of the stimuli design can be found in [4]. Because the angle and radius were chosen randomly the polygons were highly irregular (see Fig 1). For each set of vertices a cubic spline generated a curve through the vertices thus transforming the angular polygon into a smoothed version. Note how the two sets are very similar except for the smoothness of the contour.

The attribute words were selected from the ANEW database [29]. For IAT 1 (Danger) we selected 5 words for *safe* (protected, home, comfort, health, secure) and 5 words for *threat* (torture, bomb, killer, murderer, weapon). For IAT 2 (Valence) we selected 5 *positive* words (lucky, success, laughter, rainbow, paradise) and 5 *negative* words (evil, tragedy, hatred, rejected, filth). We selected words that were similar for valence: both safe and positive words had high levels of valence (M = 7.84, SD = .63) and both threatenings and negative words had low levels of valence (M = 2.20, SD = .81). Moreover they differed in the level of arousal, so that safe words had lower arousal compared to positive ones (safe: M = 4.10, SD = 2.68; positive: M = 5.83, SD = 2.75) and that threat words had higher arousal compared to negative ones (threat: M = 6.78, SD = 2.33; negative: M = 6.16, SD = 2.50). Therefore, for the Valence dimension the level of arousal was balanced, whereas for the Danger dimension we accepted that more dangerous words, by definition, had also on average higher arousal.

In Table 1 values for valence and arousal are reported for each word. For the Gender IAT we chose 64 forenames (32 male and 32 female) and we asked nine naive participants (5 female, 4 male, age range: 18–51 years) to evaluate them on three dimensions (gender: feminine-masculine; commonality: common-rare; and preference: liking-disliking) by using a visual scale. These nine individuals did not take part in the subsequent experiment. Next we used this data for selection: we relied on extreme positions on the gender dimension (extremely feminine or masculine) and middle positions on the communality and liking dimensions. We obtained 10 female names (Sophie, Hannah, Emma, Laura, Alice, Ellie, Katie, Holly, Elisabeth, Amy) and 10 male names (Joseph, Luke, Matthew, William, Joshua, Adam, Harry, Daniel, Jack, Thomas).

**Table 1:** Valence and Arousal for words selected from the ANEW database in IAT 1 and IAT 2.

WORDS	IAT 1 (Danger)				WORDS	IAT 2 (Valence)			
	Valence M	Valence SD	Arousal M	Arousal SD		Valence M	Valence SD	Arousal M	Arousal SD
***Safe***					***Positive***				
COMFORT	7.07	2.14	3.93	2.85	LAUGHTER	8.45	1.08	6.75	2.50
HEALTH	6.81	1.88	5.13	2.35	LUCKY	8.17	1.06	6.53	2.34
HOME	7.91	1.63	4.21	2.94	PARADISE	8.72	0.60	5.12	3.38
PROTECTED	7.29	1.79	4.09	2.77	RAINBOW	8.14	1.23	4.64	2.88
SECURE	7.57	1.76	3.14	2.47	SUCCESS	8.29	0.93	6.11	2.65
Total mean	**7.33**	**1.84**	**4.10**	**2.68**	Total mean	**8.35**	**0.98**	**5.83**	**2.75**
***Threat***					***Negative***				
BOMB	2.10	1.19	7.15	2.40	EVIL	3.23	2.64	6.39	2.44
KILLER	1.89	1.39	7.15	2.40	TRAGEDY	1.78	1.31	6.24	2.64
MURDERER	1.53	0.96	7.47	2.18	HATRED	1.98	1.92	6.66	2.56
TORTURE	1.56	0.79	6.10	2.77	REJECTED	1.50	1.09	6.37	2.56
WEAPON	3.97	1.92	6.03	1.89	FILTH	2.47	1.68	5.12	2.32
Total mean	**2.21**	**1.25**	**6.78**	**2.33**	Total mean	**2.19**	**1.73**	**6.16**	**2.50**

Participants sat at approximately 60cm from the screen. Stimuli were presented on an Apple StudioDisplay 21" CRT monitor (1280 X 1024 at 60Hz).

#### Experimental Design and Procedure

Each participant completed 3 IAT experiments. For the structure of each IAT experiment we followed recommendations by Nosek et al. [19].

**Fig 2:**
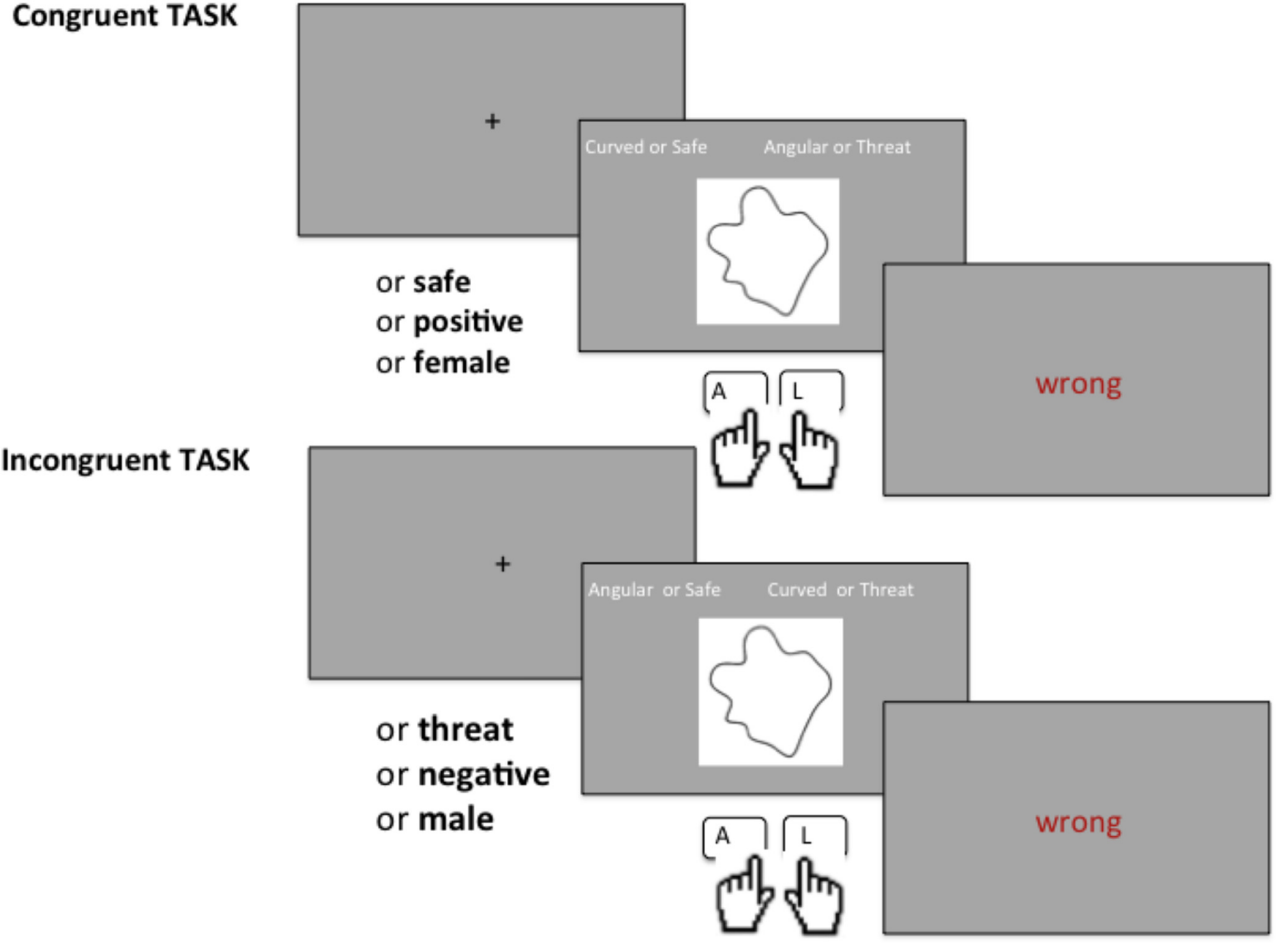
Illustration of the experimental procedure for the implicit association task (IAT) used in Experiment 1. Above each stimulus, cue words were presented on the left and right sides of the screen according to the response mapping of that trial. The message “Wrong” appeared if participants pressed the wrong button.

Each single IAT consisted of 8 blocks in total and it took 7 minutes to complete. Half the participants received the congruent blocks first (see Table 2), the other half participants received the incongruent blocks first and the training blocks were rearranged accordingly (see Table 3). To explain the sequence of events we take for example IAT 1 (Danger). In the first training block (1) participants discriminated shapes only (left key for curved, right key for angular). In the second training block (2) participants discriminated words only (left key for safe, right key for danger). Next there were two *congruent* experimental blocks (3 and 4), in which shapes (curved or angular) and words (safe or danger) were presented in alternate trials. Participants pressed one key, the same for curved shapes or safe words (left key), and another key, the same for angular shapes or danger words (right key). In this case the response mapping was congruent to our hypothesis. Next, two additional training blocks (5 and 6) were presented that included only the shapes. Here, the response mapping was reversed: participants learned to use the left key to report angular shapes, and the right key to report curved shapes. There were then other two experimental blocks (7 and 8) of *incongruent* trials where participants pressed one key, the same for angular shapes or safe words (left key), and another key, the same for curved shapes or danger words (right key). In these blocks the response mapping was opposite to our hypothesis (see the response mapping in the three IAT dimensions in Table 4). While reversed response mapping is known to impair performance in itself, the additional training blocks and feedback on each trial should minimize this order effect [16]. The shapes were presented once in each block and the order was randomized across subjects. Following the typical structure of the IAT, words and images were used as examples of categories, and were repeated across blocks. In all trials the stimuli remained on the screen until response. Above each stimulus, cue words were presented on the left and right sides of the screen according to the response mapping of that trial. For example, when an angular polygon appeared, the cues “curved” and “angular” were presented, whereas when a word appeared, the cues “safe” and “danger” were presented. If participants pressed the wrong button, the message “Wrong” appeared (see illustration of the procedure in Fig 2). Participants were instructed to make accurate responses as quickly as possible. Written instructions were presented on-screen before each block.

**Table 2:** Order of blocks and response mappings for participants who received congruent trials first (example from IAT 1).

Block		N trials	Left key	Right key
1	Training 1	20	Curved	Angular
2	Training 2	20	Safe	Threat
3	Congruent 1	20	Curved or Safe	Angular or Threat
4	Congruent 2	40	Curved or Safe	Angular or Threat
5	Training 3	20	Angular	Curved
6	Training 4	20	Angular	Curved
7	Incongruent 1	20	Angular or Safe	Curved or Threat
8	Incongruent 2	40	Angular or Safe	Curved or Threat

**Table 3:** Order of blocks and response mappings for participants who received incongruent trials first (example from IAT 1).

Block		N trials	Left key	Right key
1	Training 1	20	Angular	Curved
2	Training 2	20	Safe	Threat
3	Incongruent 1	20	Angular or Safe	Curved or Threat
4	Incongruent 2	40	Angular or Safe	Curved or Threat
5	Training 3	20	Curved	Angular
6	Training 4	20	Curved	Angular
7	Congruent 1	20	Curved or Safe	Angular or Threat
8	Congruent 2	40	Curved or Safe	Angular or Threat

**Table 4:** The response mapping in the 3 IAT dimensions.

	IAT 1 (Danger)		IAT 2 (Valence)		IAT 3 (Gender)	
	Left key	Right key	Left key	Right key	Left key	Right key
**Congruent blocks**	Curved	Angular	Curved	Angular	Curved	Angular
	Safe	Threat	Positive	Negative	Female	Male
**Incongruent blocks**	Angular	Curved	Angular	Curved	Angular	Curved
	Safe	Threat	Positive	Negative	Female	Male

#### Data analysis

We applied the same a priori criteria for excluding participants as reported in the literature [19]: trials where participants had pressed the wrong button, or where response time was > 10 s were excluded (0.4%). Subjects for whom more than 10% of trials have latencies < .03s were excluded. None of the participants was excluded from the analysis.

Adopting the same procedures for data analysis as [19, 21], training blocks were not analyzed. For each participant we calculated the mean reaction time (RT) for the congruent and incongruent trials in each IAT dimension. Descriptive statistics on RTs are included for illustrative purposes. A D score was calculated as the difference between incongruent and congruent blocks in standard deviation units. A positive value means the hypothesis was supported, and negative value means that the participant associated stimuli in the opposite way to that predicted. We obtained three D scores from each participant: one for each IAT dimension. Therefore the D score is the index of the strength of the associations between shapes and concepts. The statistical analyses were conducted on the D scores as this measure is more informative than RTs [19]. These variables were normally distributed according to the Shapiro-Wilk test (p > 0.297). The statistical analyses were conducted using SPSS (IBM Corp) [28].

### Results

**Fig 3:**
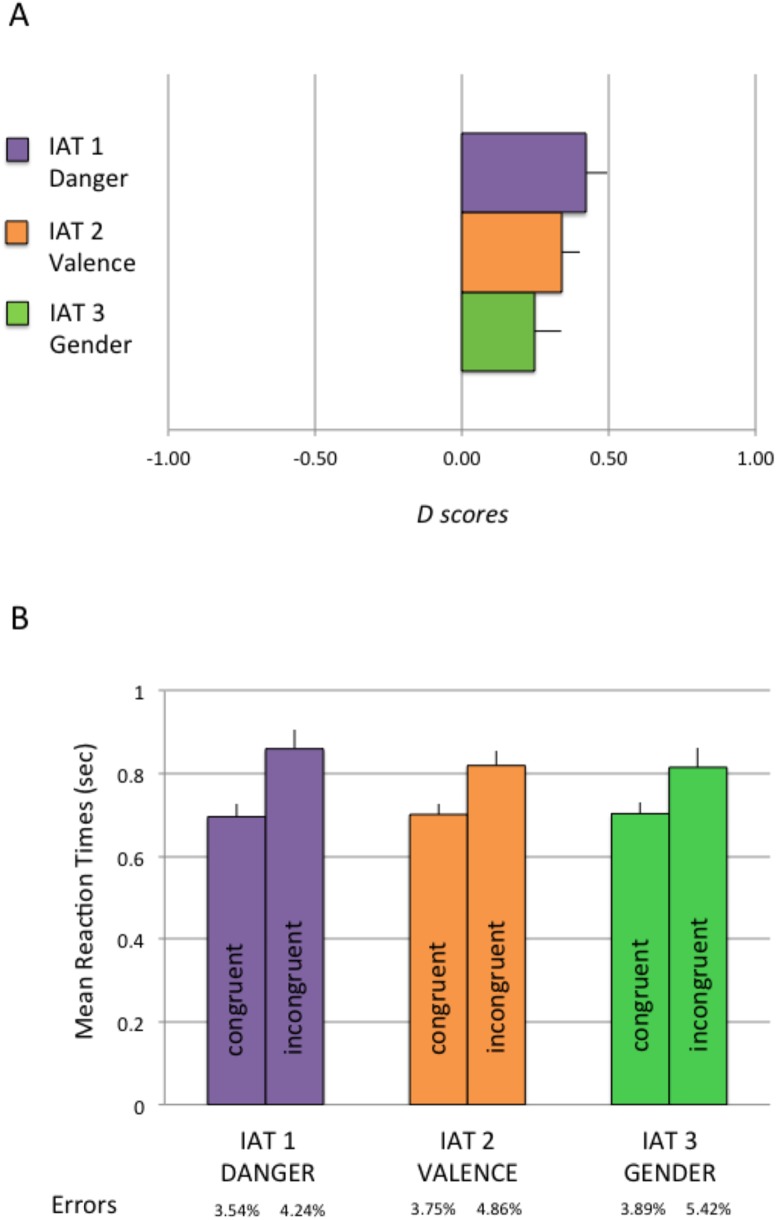
Results of Experiment 1. Panel A: *D scores* are plotted as a function of the 3 IAT dimensions (danger vs. valence vs. gender). In all three experiments there was a significant shift meaning that participants associated curved polygons to safe concepts, to positive words and to female names compared to the opposite. For angular polygons participants associated angular polygons with danger concepts, negative valence and male names. Error bars are SE of the mean. Panel B: means of RTs are shown for congruent and incongruent trials across the three IAT experiments (danger vs. valence vs. gender). Error bars are SE of the mean. At the bottom error rates are reported for congruent and incongruent trials.

Results are illustrated in Fig 3. Descriptive statistics reported similar RTs for the three IAT dimensions in the congruent trials (danger: M = .699, SD = .145; valence: M = .701; SD = .127; gender: M = .706, SD = .132) as well as in the incongruent trials (danger: M = .864, SD = .237; valence: M = .826; SD = .178; gender: M = .810, SD = .216). As expected, RTs in the incongruent trials were slower.

One-sample *t*-tests confirmed that *D scores* across participants were significantly greater than zero in the positive direction for each IAT experiment (Danger: (t(23) = 5.59, *p* = .000, Cohen’s *d* = 2.33); Valence: (t(23) = 5.41, *p* = .000, Cohen’s *d* = 2.26) and Gender: (t(23) = 2.72, *p* = .012, Cohen’s *d* = 1.13).

A 3X3X2X2 mixed measures ANOVA on D scores with IAT (danger vs. valence vs. gender) as the within-subjects factor, Experiment order (danger first vs. valence first vs. gender first), Task order (congruent first vs. incongruent first) and Gender of the participants (female vs. male) as the between-subjects factors reported no main effect of IAT (F(2, 46) = 1.70, p = .194 *η*_*p*_^*2*^ = .111), meaning that there were no significant differences between the three IAT dimensions (threat vs. valence vs. gender) on these implicit associations (danger: M = .42; SD = .37; valence: M = .34, SD = .31 and gender: M = .25, SD = .44). All the other effects were also not significant (p >.05). Finally a correlation analysis showed that the *D scores* for IAT 2 (valence) and IAT 3 (gender) correlated positively (r(23) = .418, *p* = .042). Another mixed measures ANOVA on error rates with the same factors as above reported no significant main effects (*p* >.05).

### Discussion

In Experiment 1 the three tasks showed that curved shapes were implicitly associated with safe and positive words and with female names. In contrast angular shapes were implicitly associated with danger and negative words and with male names. Therefore our hypothesis was confirmed with both female and male participants. Looking at the *D scores* the higher value resulted for the danger dimension. The strength of associations for the two dimensions of valence and gender correlated positively. However, speed for congruent trials did not differ across the three dimensions (danger vs. valence vs. gender). The results show that people tend to like curved abstract shapes because they are implicitly associated to safe and positive concepts. Instead, they tend to link angular shapes with negative concepts and threat. Unfortunately given the structure of the IAT it cannot be established whether the associations are driven more (or exclusively) by the curved shapes or by the angular shapes.

## Experiment 2: Curvature and Approach

In Experiment 2 we used the SRC task-R [4, 5]. A small stick figure that represents a person appeared on the screen after a key press. The task was to move the manikin towards or away from the shape displayed at the centre of the screen. The rationale is that if the angular shapes are perceived as threatening, then participants should move the manikin away from them (compatible trials) faster than towards them (incompatible trials). The opposite pattern was expected with curved shapes: if the curved shapes are perceived as safe and attractive then participants should move the manikin towards them (compatible trials) faster than away from them (incompatible trials).

In this experiment we used one set of irregular polygons similar to the ones employed in Experiment 1 and another set with more pronounced convexities and concavities. This task tests whether the implicit associations of concepts (e.g. safe or threat) with polygons (curved or angular) found in Experiment 1 can generate a congruent approach/avoidance response. Importantly, this task also tests whether avoidance for angular polygons is stronger than approach for curved polygons, or the opposite way around.

### Method

#### Participants

Thirty-six participants took part (age range: 18–38; 3 left handed; 30 females). All participants had normal or corrected to normal vision and provided written consent. The experiment received ethics approval and was conducted in accordance with the Declaration of Helsinki (2008).

#### Stimuli and apparatus

Stimuli consisted of irregular abstract polygons similar to those used in Experiment 1. A full explanation of the stimuli design can be found in [4]. Each shape was generated using 22 vertices starting from a circle. Two sets of polygons were generated by varying the minimum and maximum values for the radius. In one case the values were between 80 and 120 (range 40) and in the other case the values were between 60 and 140 (range 80).

The manikin consisted of a stick figure (2.5 cm high ad 1 cm wide) with a circle for the head and straight lines for the body and limbs. During movement the right leg and the left leg became longer and shorter to generate the impression of walking. Participants sat at approximately 60cm from the screen. Stimuli were presented on an Apple StudioDisplay 21" CRT monitor (1280 X 1024 at 60Hz).

#### Experimental Design and Procedure

**Fig 4:**
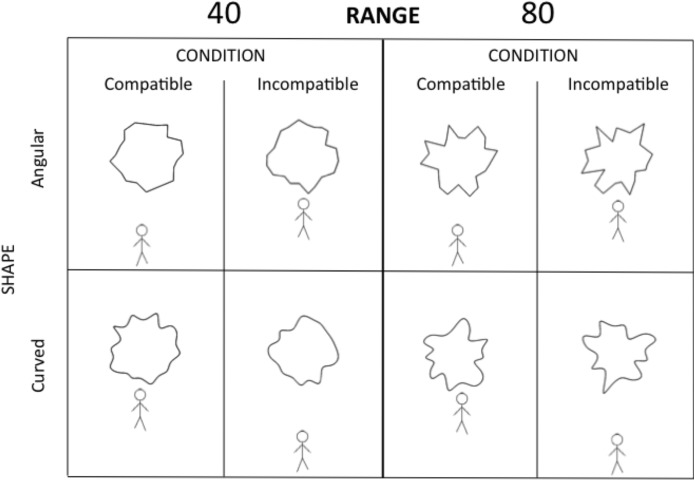
Experiment 2: Illustration of the experimental design. The left panel shows the two tasks (compatible vs. incompatible) with stimuli range 40. The right panel shows the two tasks (compatible vs. incompatible) with stimuli range 80. In the compatible task the manikin was moved by the participant away from an angular shape (top) or towards a curved shape (bottom). In the incompatible task the manikin was moved by the participant towards an angular shape (top) and away from a curved shape (bottom). In this figure the manikin is shown always underneath the stimulus, but it was presented above the stimulus with equal probability.

A 2 x 2 x 2 within-subjects design was employed with as factors: Condition (compatible vs. incompatible); Shape (angular vs. curved) and Range (40 vs. 80). A trial started with a fixation cross. Participants were instructed to press 5 on the numeric pad to let the manikin appear on the bottom or on the top of the screen. After 750 ms a shape (angular or curved) was presented at the centre of the screen. Following the original task [18] as well as our previous application [4, 5] participants were instructed to move the manikin as quickly and accurately as possible by pressing 8 (upward) or 2 (downward) on the numeric pad. Depending on the initial position of the manikin (bottom or top) and the movement direction (upward or downward), the figure stopped either at the edge of the screen or close to the shape (Fig 4). In our version of the SRC task [4, 5] participants needed to press the same key three times to move the figure (38 pixels per step) to the end position. This approach has been previously adopted to give the impression that the manikin was walking and moving in steps [25]. The screen turned black 50 ms after the third key-press. Participants completed one compatible block of 96 trials and one incompatible block of 96 trials. Each block was preceded by 8 practice trials. The order of the compatible and incompatible blocks was counterbalanced across participants, and within a block trials were presented in random order. The experiment lasted approximately 40 minutes.

#### Analysis and data reduction

A 2x2x2 repeated measures ANOVA was performed for RT with Condition (compatible vs. incompatible) Shape (angular vs. curved) and Vertices (range 40 vs. range 80) as within-subjects factors. The dependent measures were RTs and error rates.

Differently from all previous versions [18, 25, 4, 5], here we analysed two measures: RT1, which reflects the time elapsed between the appearance of the stimulus and the first key press; and RT3, which reflects the time elapsed between the appearance of the stimulus and the third key press (when the manikin reached the edge of the screen or the shape), namely the combination of simple RT and the time required to completing the ballistic movement of response execution [26, 27]. Only RTs data for which participants pressed the correct key were analyzed. One participant was excluded from the analysis on the RTs because he/she made errors in more than 25% of the trials, which made it not possible to compute the mean for each condition. The statistical analyses were conducted using SPSS (IBM Corp) [30].

## Results

**Fig 5:**
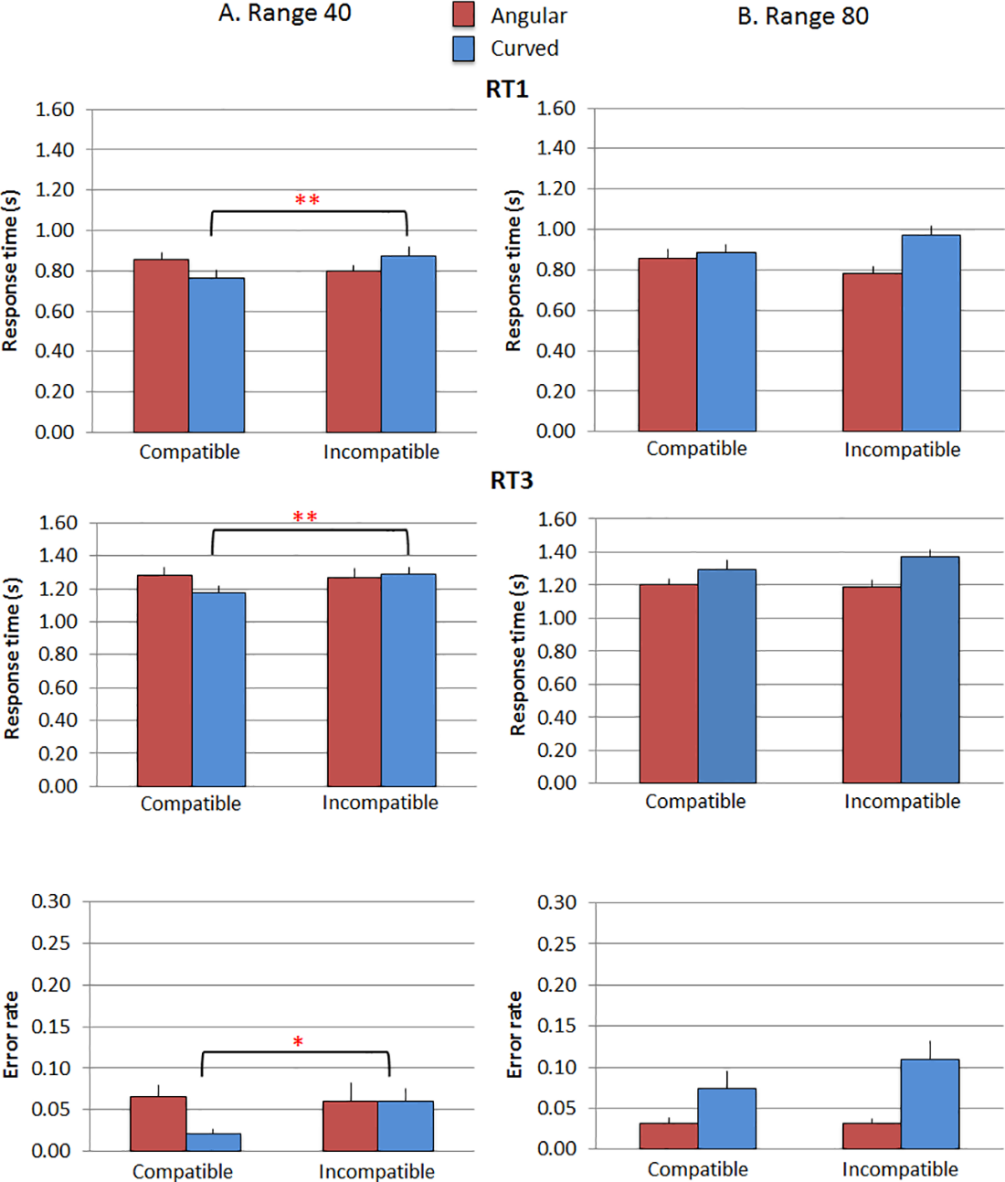
Results of Experiment 2. (A) stimuli with vertices range 40, (B) stimuli with vertices range 80. Top quadrant: RT1, participants’ RTs (y axis) as a function of Condition (compatible vs. incompatible; x axis) and Shape (angular vs. curved; separate bars). Middle quadrant: RT3, participants’ RTs (y axis) as a function of Condition (compatible vs. incompatible; x axis) and Shape (angular vs. curved; separate bars). Bottom quadrant: participants’ error rates (y axis) Condition (compatible vs. incompatible; x axis) and Shape (angular vs. curved; separate bars). Error bars are SE of the mean.

The results are illustrated in Fig 5.

The ANOVA on RT1 reported a main effect of Shape (F(1, 35) = 13.56, *p* = .001 *η*_*p*_^*2*^ = .279). Participants overall performed faster with angular polygons (M = .82; SD = .20) rather than with curved ones (M = .87; SD = .21). Importantly, the interaction effect of Condition by Shape was significant (F(1, 35) = 11.27, *p* = .002 *η*_*p*_^*2*^ = .244). This revealed that RTs differed between compatible and incompatible trials with curved polygons (t(34) = -3.05, *p* = .004, Cohen’s *d* = -1.05) but not with angular polygons (t(34) = .302, *p* = .765, Cohen’s *d* = .10). Participants did not move the manikin away from the angular polygons faster (M = .83; SD = .19) than towards them (M = .82; SD = .25). In contrast participants were faster to move the manikin closer to the curved polygons (M = .82; SD = .24) than away from them (M = .93; SD = .24). There was also a significant interaction effect of Shape by Vertices (F(1, 35) = 37.80, *p* = .000 *η*_*p*_^*2*^ = .519), meaning that RTs were faster for curved polygons with less pronounced vertices (range 40) and for angular polygons with more pronounced vertices (range 80). The three-way interaction Condition by Shape by Vertices was not statistically significant (F(1, 35) = .003, *p* = .954 *η*_*p*_^*2*^ = .000).

The results of the ANOVA on RT3 overlapped those on RT1, although with a weaker effect size. A main effect of Shape (F(1, 35) = 10.42, *p* = .003 *η*_*p*_^*2*^ = .229) was found: participants performed faster with angular polygons (M = 1.24; SD = .24) rather than with curved ones (M = 1.27; SD = .23). Importantly, the interaction effect of Condition by Shape was also significant (F(1, 35) = 8.66, *p* = .006 *η*_*p*_^*2*^ = .198): RTs differed between compatible and incompatible trials with curved polygons (t(34) = -2.207, *p* = .034, Cohen’s *d* = .75) but not with angular polygons (t(34) = .345, *p* = .732, Cohen’s *d* = .12). Moreover, participants were faster for curved polygons with less pronounced vertices (range 40) and for angular polygons with more pronounced vertices (range 80) as reflected by the significant interaction effect of Shape by Vertices (F(1, 35) = 32.20, *p* = .000 *η*_*p*_^*2*^ = .479).

A similar pattern of results was found for the error rates. The number of errors for the angular stimuli did not differ between compatible and incompatible trials (angular polygons range 40: t(35) = .223, *p* = .825, Cohen’s *d* = .07; angular polygons range 80: t(35) = .000, *p* = .100, Cohen’s *d* = .00). In contrast for the curved polygons in the condition where they were more rounded (range 40) there were fewer errors in the compatible relative to the incompatible condition (t(35) = -2.53, *p* = .016, Cohen’s *d* = .86). In the condition where curved polygons presented more pronounced vertices (range 80) the difference was not significant (t(35) = -1.82, *p* = .077, Cohen’s *d* = .62), although the trend was in the same direction. Overall participants made fewer errors when moving the manikin towards the curved polygons than away from them.

### Discussion

In Experiment 2 we found that angular polygons do not generate an avoidance response: participants were faster for the angular shapes as compared to the curved shapes in both conditions (towards and away). Interestingly, this pattern of result was found for angular polygons with less pronounced vertices (range 40) as well as with angular polygons where the vertices appeared sharper (range 80), and therefore potentially more threatening.

We found that moving the manikin towards curved polygons was faster and less effortful than moving it away from them. The use of two different ranges revealed that faster responses were made with curved polygons appearing more rounded (range 40). Therefore, overall these findings favour an approach response towards the curved polygons, rather than an avoidance response for the angular polygons. In conclusion the results of Experiment 2 support the hypothesis that the preference for smoothly curved polygons is not exclusively a by-product of the avoidance for angular polygons [10], and that there is an approach response to smooth curvature.

## General Discussion

During the last decade the origin of the preference for curved contours has become a central question in empirical aesthetics as well as in related applied fields [3, 4, 5, 6, 7, 10, 13]. This might be because the contour conveys important information about a shape, and variations in the amount of angularity/roundness have a strong impact on object classification and in preference formation.

One proposal is that angles signal threat [3, 10, 12]. However, it has also been argued that there is a positive response to smooth curvature [5, 6, 13]. These studies did not establish whether one of the two responses (threat for angles or reward for curvature) plays the crucial role in the preference for curvature. Moreover they only used explicit measures to investigate the link between visual preference for shapes and affective reactions.

The current work was focused on one specific aspect related to the formation of preference for curved over angular shapes: the underlying semantic/affective processes which might be implicitly associated with the stimuli.

### The link between preference for curved shapes and implicit responses

The current study had reached three aims: To verify the presence of implicit associations between shapes contour line (curved vs. angular) and different attributes (valence, danger and gender). If these associations exist then this would suggest that the liking response for abstract shapes is interconnected to the meaning they implicitly recall (Experiment 1). The second aim was to verify whether these implicit associations would trigger a congruent affective (approach/avoidance) reaction and whether the avoidance of angular shapes would be stronger than the approach of curved shapes, or the other way around. This would clarify whether preference for curvature is a by-product of the dislike for angularity or whether curvature is preferred per se (Experiment 2). Third, the use of two implicit measures that complement each other could reveal more information about the semantic/affective processes underlying preference as compared to explicit liking evaluations (Experiment 1 and 2).

We designed irregular polygons with angular or curved contour lines, matched for all other aspects (number of vertices, size, orientation). This allowed the control of familiarity and semantic processes, which typically influence liking evaluations.

The Implicit Association test [14] allowed us to measure the strength of the association between target stimuli (in our case the curved or angular contour line of irregular polygons) and different categories (e.g. positive or negative valence). The IAT has been extensively used as a tool to investigate implicit preference for different classes of stimuli [20, 21, 22, 23, 24]. In Experiment 1 we employed a multidimensional IAT to examine implicit associations between angular and curved shapes and attributes: IAT 1: danger (safe-threat), IAT 2: valence (positive-negative) and IAT 3: gender (female-male).

As predicted, curved shapes were implicitly associated with safe and positive words and with female names. In contrast angular shapes were associated with danger and negative words and with male names. The response latency for congruent trials did not differ across the three dimensions (Danger vs. Valence vs. Gender). The results of Experiment 1 can indicate, although indirectly, that observers tend to like curved abstract shapes because they are implicitly associated with safe and positive concepts and because they recall female attributes. To the same extent observers tend to dislike angular shapes for their link to negative concepts or threat. Therefore the IAT support the hypothesis that the liking response for abstract shapes is also linked to associations with other dimensions. However, the IAT alone cannot determine whether the associations found with curved shapes are stronger than the associations found with angular shapes.

In Experiment 2 we used a revised version of the SRC task [18, 4, 5] to test implicit approach-avoidance reactions to curved and angular polygons respectively. Similarly to the IAT, this task can be seen as an indirect measure of preference, or at least as a tool to investigate implicit affective reactions underlying preference formation. The current SRC task-R introduced a different approach to analyse the data, which consisted of taking into account both RT1 and RT3, measuring respectively how fast participants decided whether to approach or avoid the shape and how fast they kept the manikin moving in that direction. Results clearly showed the presence of an approach reaction with curved shapes and the absence of an avoidance reaction with angular shapes, even when the polygons showed sharper vertices. Therefore, the hypothesis that curvature is liked as a consequence of the dislike for angularity is not confirmed. Rather, it is the curved appearance of the contour that made the polygons liked more [4, 5].

Interestingly, our results with the SRC task-R do not match previous findings on the approach-avoidance dimension investigated with explicit evaluations [13]. This is possibly due to the fact that the involvement of decision-making operations can mediate or mask the impact of affective dimensions of stimuli in the formation of the liking response.

## Conclusion

The curvature effect can be explained (1) as a by-product of the dislike for angularity (angles are associated to threat) [10]; or (2) by a genuine visual preference for curvature, because of its configuration (or gestalt) [4, 5]; or (3) by the combination of the two: both (implicit) associations and visual processing might play a role but at a different level of the processing stage. The current work established that there are implicit associations of shapes with different semantic dimensions, for example curved shapes with words expressing “safe” concepts and angular shapes with words expressing “danger” concepts. However, Experiment 1 did not test which one of the two associations is stronger. Our SRC task-R in Experiment 2 showed that only curved shapes generated an approach reaction. In this respect our SRC task-R was more informative than the IAT. Moreover, it revealed affective processes underlying preference for curvature, which might not be accessible with explicit evaluations. We suggest that preference for curved contours might be triggered by an aesthetic quality contained in the curved contour itself, but it is also mediated by what curves can recall in terms of affective representations. Possibly the visual preference for curves is boosted by positive feedbacks from second-order associative processes. Further investigations are needed to clarify how these processes interact.
